# Copeptin reflects physiological strain during thermal stress

**DOI:** 10.1007/s00421-017-3740-8

**Published:** 2017-10-27

**Authors:** Michael John Stacey, Simon K. Delves, Sophie E. Britland, Adrian J. Allsopp, Stephen J. Brett, Joanne L. Fallowfield, David R. Woods

**Affiliations:** 1 Department of Surgery and Cancer, Imperial College London, Care of General Intensive Care Unit, Hammersmith Hospital Campus, Du Cane Road, London, W12 0HS UK; 2 Department of Military Medicine, Royal Centre for Defence Medicine, ICT Building, Birmingham Research Park, Vincent Drive, Edgbaston, Birmingham, B15 2SQ UK; 3 Institute of Naval Medicine, Alverstoke, Hampshire PO12 2DL UK; 4 Carnegie Research Institute, Leeds Beckett University, Leeds, LS6 3QS UK

**Keywords:** Dehydration, Heat illness, Acute kidney injury, Arginine vasopressin, Normetanephrine, Cortisol

## Abstract

**Purpose:**

To prevent heat-related illnesses, guidelines recommend limiting core body temperature (*T*_c_) ≤ 38 °C during thermal stress. Copeptin, a surrogate for arginine vasopressin secretion, could provide useful information about fluid balance, thermal strain and health risks. It was hypothesised that plasma copeptin would rise with dehydration from occupational heat stress, concurrent with sympathoadrenal activation and reduced glomerular filtration, and that these changes would reflect *T*_c_ responses.

**Methods:**

Volunteers (*n* = 15) were recruited from a British Army unit deployed to East Africa. During a simulated combat assault (3.5 h, final ambient temperature 27 °C), *T*_c_ was recorded by radiotelemetry to differentiate volunteers with maximum *T*_c_ > 38 °C versus ≤ 38 °C. Blood was sampled beforehand and afterwards, for measurement of copeptin, cortisol, free normetanephrine, osmolality and creatinine.

**Results:**

There was a significant (*P* < 0.05) rise in copeptin from pre- to post-assault (10.0 ± 6.3 vs. 16.7 ± 9.6 pmol L^−1^, *P* < 0.001). Although osmolality did not increase, copeptin correlated strongly with osmolality after the exposure (*r* = 0.70, *P* = 0.004). In volunteers with maximum *T*_c_ > 38 °C (*n* = 8) vs ≤ 38 °C (*n* = 7) there were significantly greater elevations in copeptin (10.4 vs. 2.4 pmol L^−1^) and creatinine (10 vs. 2 μmol L^−1^), but no differences in cortisol, free normetanephrine or osmolality.

**Conclusions:**

Changes in copeptin reflected *T*_c_ response more closely than sympathoadrenal markers or osmolality. Dynamic relationships with tonicity and kidney function may help to explain this finding. As a surrogate for integrated physiological strain during work in a field environment, copeptin assay could inform future measures to prevent heat-related illnesses.

## Introduction

Individuals who perform strenuous work are at risk of adverse effects associated with excessive body heat (‘heat illness’), varying from physical incapacity to severe injury and death (Armstrong et al. 2007). A widely accepted definition of heat stroke is as ‘a form of hyperthermia associated with a systemic inflammatory response, leading to a syndrome of multiorgan dysfunction in which encephalopathy predominates’ (Leon and Bouchama 2015).

To minimise the incidence of heat illness, occupational guidelines recommend that core body temperature (*T*_c_) is maintained at or below 38.0 °C. Despite these guidelines, *T*_c_ exceeds 38.0 °C during a number of occupational activities (Meade et al. 2016). There are considerable practical limitations to rectal or intra-gastric *T*_c_ monitoring and sampling at the tympanic membrane may be unhelpful, due to lack of uniformity of the temperature response (Yeoh et al. 2017) and the potential for hearing and thus safe working to be affected. Furthermore, the inter-individual response to thermal stress is diverse; the exact significance of core temperature in the pathophysiology of heat stroke remains debated (Noakes 2008); and evidence from civilian occupational settings (Garzon-Villalba et al. 2016) and military training (Wallace et al. 2005) indicates a cumulative effect of prior-day thermal stress on risk of heat-related illnesses. Thus instantaneous *T*_c_ values may incompletely reflect the risk of heat illness. A marker that integrates the physiological strain incurred during thermal stress, including but not limited to *T*_c_ response, could prove useful in prospectively identifying which individuals exposed to the same working conditions will become casualties.

The posterior pituitary hormone arginine vasopressin (AVP) classically rises in response to hypovolaemia and increasing osmolality, and is secreted as a large peptide precursor ProAVP. ProAVP consists of AVP, copeptin, neurophysin II and a signal peptide. With exertion in a warm environment, AVP has been shown to relate closely to plasma osmolality (Montain et al. 1997), which is a preferred blood marker of dehydration from thermal sweating (Cheuvront and Kenefick 2014). Thus an index of AVP secretion could usefully reflect physiological strain under thermal stress.

The assay of AVP itself is highly problematic (it is labour intensive, requires multiple pre-analytical steps, has a half-life < 30 min and is unstable even in isolated plasma), therefore recently copeptin (which is stable and can be measured on an automated analyser) has found favour as a valid and practical surrogate for AVP secretion (Morgenthaler 2012; Christ-Crain and Fenske 2016). Copeptin secretion occurs in response to various stressors (Christ-Crain and Fenske 2016; Katan et al. 2008a, b) and its concentration in peripheral blood has been proposed as a sensitive marker of the individual stress level (Katan et al. 2008a). In healthy subjects performing a high altitude trek, plasma concentrations of copeptin and AVP associated closely and the correlation was strengthened (*r* = 0.834, *p* < 0.001) at the highest and most stressful measurement point (Mellor et al. 2015). In disease, copeptin has been found to be of prognostic value in diverse conditions such as sepsis (Seligman et al. 2008), septic and haemorrhagic shock (Morgenthaler et al. 2007), lower respiratory tract infections (Müller et al. 2007), cardio- and cerebrovascular disease (Sun et al. 2015; Stoiser et al. 2006; Marston et al. 2016; Greisenegger et al. 2015) and for end stage renal failure, coronary heart disease and all-cause mortality in patients with type 1 diabetes (Velho et al. 2016).

One important factor that may contribute to AVP/copeptin release is body temperature. Enhanced secretion of AVP with increasing tissue temperature has been demonstrated both in resting human subjects (Takamata et al. 1995a) and in hypothalamic-pituitary explants warmed from 37.0 to 39.5 °C (Sladek and Johnson 2013), whereby the rise in AVP was additive with that from increasing osmolality of plasma/perfusate. The effect of *T*_c_ on copeptin concentration is yet to be investigated, however, and relationships of copeptin with other parameters of physiological strain under thermal stress have not been characterised. We hypothesised that plasma copeptin would rise with dehydration from occupational heat stress, concurrent with serum osmolality and creatinine (sCr), and that changes in copeptin would reflect *T*_c_ response. As a *T*_c_ of 38.0 °C marks an important thermoregulatory threshold during exertional heat stress (Commission for Thermal Physiology 1987) above which vasomotor compensation plateaus (Gonzalez-Alonso et al. 1999) and sensitive control of *T*_c_ is lost (Wyndham et al. 1965) we chose a *T*_c_ of 38.0 °C as a threshold for the initial investigation of copeptin in response to thermal stress. As sympathoadrenal activation is augmented by *T*_c_ rise (Rhind et al. 1999), we additionally investigated changes in cortisol and free normetanephrine levels as alternative markers of physiological strain under heat stress.

## Methods

### Ethical approval

This study was approved by the United Kingdom (UK) Ministry of Defence Research Ethics Committee (465/MODREC/13) and complied with the standards set in the Declaration of Helsinki. All volunteers gave written informed consent.

### Study design

#### Preliminary measures

Volunteers were recruited to the study from a British Army infantry battalion who took part in an overseas military exercise in northern Kenya (East Africa). A total of fifteen volunteers completed study measures in the deployed field environment.

Prior to departure, volunteers underwent baseline measurements at their home duty station in the UK. A digital scale and stadiometer (SECA Ltd, Leicester, UK) were used to determine body mass, to the nearest 0.01 kg, and height, to the nearest 0.1 cm, while wearing the clothing ensemble to be worn in Kenya, minus boots. Body fat as a percentage of body composition was determined by segmental multi-frequency bioimpedance (Tanita UK Ltd., Yiewsley, Middlesex, UK).

#### Deployed measures

The volunteers deployed to Kenya 3 weeks after baseline measurements, where they undertook low-level activities in cooler upland environs. Environmental conditions were monitored locally using a wet bulb globe temperature (WBGT) monitor (Grant, Cambridge, UK) throughout. Average WBGT was 16 °C during this initial phase, which lasted for 3 weeks. They then re-located to a warm lowland environment to take part in simulated combat assaults conducted over varying terrain while on foot, with live firing of munitions. Volunteers wore full combat dress and carried an equipment load of 38.9 ± 5.0 kg. The WBGT during this phase was 26 ± 5 °C.

Deployed study measures were made in relation to a simulated combat assault on the morning of the sixth day of training in the warm environment, 6 weeks following the UK measures. Volunteers rose from overnight resting at 2.00 a.m. local time and underwent study measures in a 1 h sampling window (2.30 a.m.–3.30 a.m.). They were weighed by digital scale, to the nearest 0.01 kg (SECA Ltd, Leicester, UK), provided pre-assault (PRE) blood samples and had a plastic sweat-collection pouch applied to the left upper arm. Volunteers were then transported by vehicle to the training area, where they rested (5.00 a.m.–7.00 a.m.) until moving into position for the start of the assault (7.00 a.m.–7.30 a.m.). The field assault lasted for 3.5 h, from 8.00 a.m. to 11.30 a.m. It consisted of a staggered advance on foot into a series of ‘enemy’ defensive positions in a mock-up village, with intermittent static periods of co-ordinated weapon firing, re-grouping and re-supply (including drinking water). Post-assault (POST) blood samples were drawn after the conclusion of the assault, between 30 and 60 min following cessation of strenuous exertion (12.00 p.m.–12.30 p.m.). Where still intact, sweat was sampled from the collection pouch. Volunteers were allowed to drink water only, from rising until the completion of POST measures (2.00 a.m.–12.30 p.m.).

For *T*_c_ monitoring, each volunteer was provided with two telemetric pills (VitalSense, Mini Mitter Company Inc, Oregan, USA) to ingest 12 and 2 h before the field assault (6.00 p.m. and 6.00 a.m.). This was with the intention of using measurements from the pill that had been in the gastrointestinal tract for the longest period, in order to reduce confounding from the ingestion of water during the assault, while allowing for the possibility that the first ingested pill could be excreted before the end of the monitoring period. These transmitted wirelessly to a receiver (VitalSense, Mini Mitter Company Inc, Oregan, USA) carried on the volunteer’s person, which logged *T*_c_ locally every 60 s during the field assault. A member of each sub-unit within the group (*n* = 4) carried a Global Positioning System (GPS) (Sportslog, UK), which also logged locally every 60 s.

#### Biochemical sampling and processing

Blood was obtained by venesection in rested volunteers in a seated position from an antecubital fossa vein. Blood was drawn into in a serum separator tube (SST) (cortisol, osmolality and creatinine) and EDTA tube (plasma copeptin and free normetanephrine). Samples were stored in ice and centrifuged within 1 h. With sweat samples, they were then immediately frozen to −20 °C and maintained at this temperature during transportation back to the UK, where they were stored at −80 °C until analysis. All measurements were performed in duplicate. Copeptin was assayed using an automated sandwich immunofluorescent assay based on TRACE technology (Brahms CT-proAVP Kryptor Compact Plus, Hennigsdorf, Germany). This assay has a coefficient of variation (CV) of 2.5–3.7% and a lower limit of detection of 0.9 pmol L^−1^. Cortisol was measured using the Roche Elecsys Cortisol I assay, an automated competitive chemiluminescence immunoassay (CLIA), using the Roche modular *E* unit (Roche Diagnostics, Burgess Hill, UK). The analytical range of the assay is 0.5–1750 nmol L^−1^ and the intra-assay CV is 9.3–11.7%. Free normetanephrine was measured using an in-house liquid chromatography/tandem mass spectrometry method (CV 4–12%). Osmolality was measured using a depression of freezing point method (Advanced^®^ Model 3320 Micro-Osmometer, Advanced Instruments, Norwood, MA, USA) with a CV of 1.1%. Creatinine was measured using the Jaffe method (CV 1.7–2.3%). Sweat sodium concentration was measured by an automated indirect ion selective electrode (ISE) method, with a lower limit of quantification of 20 mmol L^−1^ and an inter-assay CV of 1.7–4.6%.

### Sample size

A power calculation was not performed prospectively as the sample size was dictated by the availability of equipment and personnel during a busy infantry exercise in a remote environment, in the face of significant logistic challenges. It was noted, however, that Morgenthaler et al. (2006) showed a significant, near two-fold increase in copeptin concentrations in a sample of 12 volunteers performing bicycle exercise (ambient conditions and *T*_c_ response not specified) and that significant elevations in AVP and osmolality were demonstrated in ten dehydrated subjects performing treadmill-walking while deprived of fluid intake over 90 min in the heat (Maresh et al. 2004). No rise in osmolality was shown when same ten subjects performed the protocol in the euhydrated state while taking water ad libitum. Thus a minimum sample size of 12 volunteers was considered adequate to mitigate the occurrence of type 2 statistical errors in respect of changes in copeptin and osmolality.

### Statistical methods

Statistical calculations were performed using the software package GraphPad Prism (GraphPad Prism version 5.01 for Windows, GraphPad Software, San Diego California USA). Parametric or non-parametric statistical tests were applied after exploring the data for normality using the Shapiro–Wilk test. Changes in two-group paired continuous data before and after the assault were analysed by Student’s paired *t* test or Wilcoxon signed ranks test for parametric and non-parametric data, respectively. For unpaired two-group comparisons of parametric and non-parametric data an unpaired *t* test and a Mann–Whitney test were used, respectively. Relationships between copeptin, osmolality and normetanephrine responses were assessed using Pearson’s (parametric data) and Spearman’s (non-parametric data) coefficients. For the reasons outlined above, *T*_c_ of 38.0 °C is a thermal threshold with physiological and occupational relevance, so an exploratory analysis was performed for the effects of the field assault (PRE to POST) against *T*_c_ group using two-way repeated measures analysis of variance (ANOVA). Sidak’s multiple comparisons test was applied where appropriate. Individuals were assigned to *T*_c_ group according to whether the maximum temperature attained during the assault was > 38.0 °C (GT38) or ≤ 38.0 °C (LT38). The effect of *T*_c_ group on each biochemical parameter was expressed as Cohen’s *d* = (mean change_GT38_ −mean change_LT38_)/pooled standard deviation (SD), where pooled SD = √((SD_GT38_^2^ + SD_LT38_^2^)/2). Thermal (temperature–time) curves were also plotted, taking a normal resting *T*_c_ of 37 °C as the baseline above which the area under the curve (AUC-Tc37) was calculated. Data are presented as mean ± SD, except where specified. Data on *T*_c_ response are provided for the telemetric pill that had been in situ for the longest period.

## Results

### Volunteer characteristics

Pre-deployment (UK) measures were as follows: age 25 ± 5 years; height 1.79 ± 0.07 m; weight 77.5 ± 8.9 kg; body mass index 25.3 ± 2.1 kg m^2^. body fat 16.2 ± 3.3%.

### Assault exposure

The ambient (dry bulb) temperature measured local to the training area increased from 20 to 27 °C during the hours of the simulated combat assault. The median distance covered from GPS measurements was 1781 (range 585–2265) m. The median moving speed was 1.3 km h^−1^ (range 0–5.8 km h^−1^).

### Core temperature

**Fig. 1:**
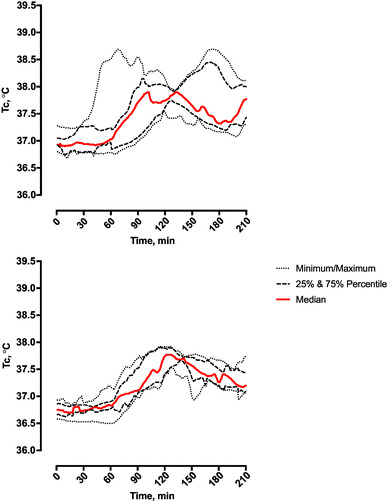
Core temperature (*T*_c_) response recorded during simulated field assault, 8.00 a.m.–11.30 a.m. Top: 8 volunteers with maximum core temperature (*T*_cMax_) greater than 38 °C during the assault (GT38). Bottom: 7 volunteers with *T*_cMax_ no greater than 38 °C (LT38)

Figure 1 presents the group *T*_c_ response before, during and after the field assault. Group *T*_c_ ranged from a minimum value (*T*_cMin_) of 36.8 ± 0.2 °C to a maximum value (*T*_cMax_) of 38.1 ± 0.4 °C, a rise of 1.3 °C (95% CI 1.2–1.5 °C, *P* < 0.0001). The highest individual *T*_c_ recorded was 38.7 °C and the greatest individual rise in *T*_c_ was 1.9 °C. Eight volunteers attained *T*_cMax_ ≥ 38.0 °C, spending 48 ± 20 min at or above this threshold, whereas *T*_c_ remained ≤ 38.0 °C in seven volunteers. The rise in *T*_c_ for GT38 (1.5 ± 0.3 °C) was higher compared with the LT38 group (1.2 ± 0.1 °C, 95% CI 0.1–0.6 °C for the difference in maximal *T*_c_ change between GT38 and LT38).

### Body mass

Clothed body mass decreased from 77.1 ± 8.2 kg PRE to 75.9 ± 8.1 kg POST (95% CI −1.69 to −0.66 kg for the difference in clothed body mass). In GT38 vs. LT38, the change in clothed body mass did not differ in either absolute (−1.4 ± 1.0 kg vs. −0.9 ± 0.8) or relative (−1.8 ± 1.4 vs. −1.2 ± 1.1%) terms.

### Biochemical results

Between PRE and POST, there was a rise in copeptin (10.0 ± 6.3 vs. 16.7 ± 9.6 pmol L^−1^, *P* < 0.001), normetanephrine (350 ± 157 vs. 576 ± 169, *P* < 0.0001) and sCr (98 ± 11 vs. 104 ± 15, *P* < 0.005), whereas cortisol decreased (620 ± 132 vs. 323 ± 125, *P* < 0.0001) and plasma osmolality did not change (291 ± 4 vs. 292 ± 4, *P* = 0.6074). Sweat sodium POST was 37 ± 12 mmol L^−1^.

**Fig. 2:**
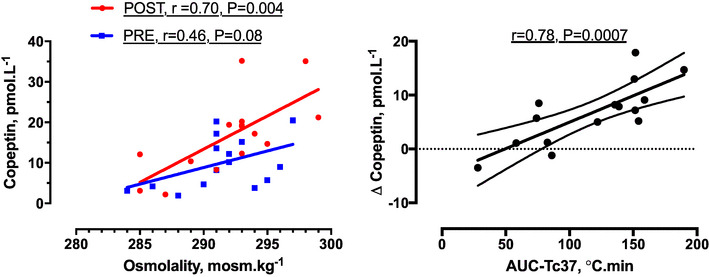
Relationships of copeptin to osmolality (left: measured before, PRE, and after, POST, exposure) and area under the core body temperature–time curve (AUC-Tc37, right: baseline *T*_c_ 37 °C, measured during the exposure and related to PRE to POST change in copeptin)

An association between copeptin and osmolality PRE (*r* = 0.46, *P* = 0.08) was strengthened POST (*r* = 0.70, *P* < 0.01; Fig. 2). The change in copeptin from PRE to POST (Δ copeptin) correlated positively with Δ creatinine, whereas its showed no significant association with Δ cortisol, Δ normetanephrine or Δ osmolality. There was also a strong correlation between Δ copeptin and AUC-Tc37 (*r* = 0.78, *P* < 0.01; Fig. 2) but no significant association between Δ copeptin and Δ body mass (Table 1).

**Table 1:** Association of Δ copeptin (PRE to POST) with corresponding changes in other parameters among 15 volunteers undergoing simulated field assault

	*r*	*P* value
Maximum Δ *T*_c_ (°C)	0.47^a^	0.08
AUC-Tc37 (°C min^−1^)	0.78^b^	< 0.01
Body mass (kg)	−0.38^b^	0.16
Δ Cortisol (nmol L^−1^)	0.40^b^	0.14
Δ Normetanephrine (pmol L^−1^)	0.30^a^	0.27
Δ Osmolality (mosm kg^−1^)	0.40^b^	0.14
Δ Creatinine (μmol L^−1^)	0.61^b^	0.02

*T*_c_ core temperature, *AUC-Tc37* area under the curve (temperature–time), baseline *T*_c_ = 37 °C

^a^Spearman *r*

^b^Pearson *r*

**Fig. 3:**
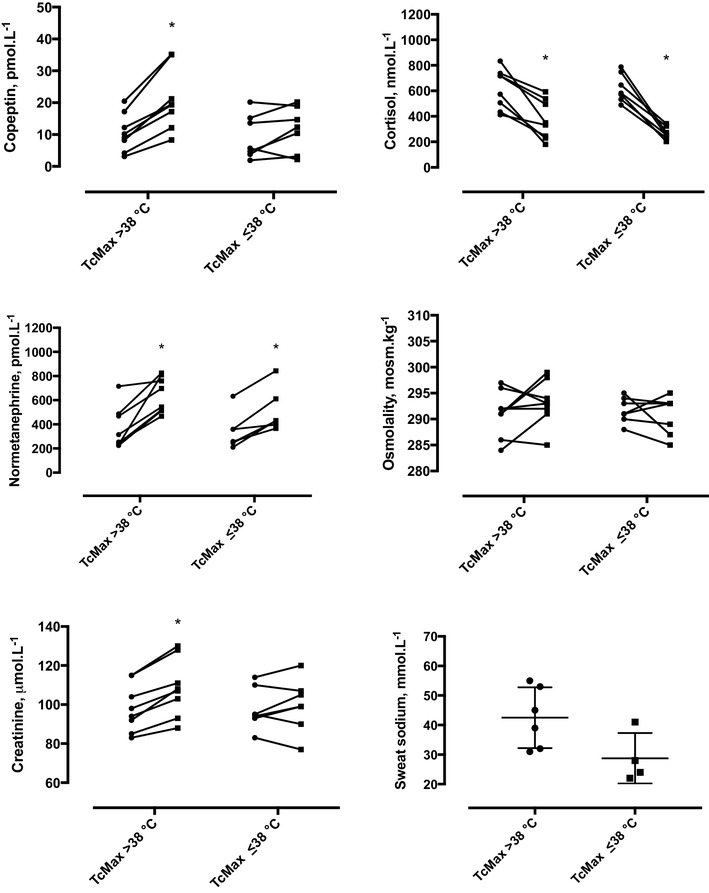
Responses in GT38 and LT38 groups, for plasma copeptin, serum cortisol, plasma normetanephrine, serum osmolality and serum creatinine (before, PRE, and after, POST, field assault) and sweat sodium (POST assault). *Significant difference PRE to POST (*P* < 0.05), by Holm–Sidak multiple comparisons test

From two-way ANOVA for repeated measures (Time by *T*_c_), significant interactions existed for changes in copeptin (interaction *F* = 13.09, *P* < 0.05) and sCr (*F* = 9.7, *P* < 0.01), but not for cortisol, normetanephrine or osmolality (Fig. 3). For GT38 only, significant changes were observed for Δ copeptin (GT38 vs. LT38: 10.4 vs. 2.4 pmol L^−1^, 95% CI of difference 6.6 to 14.2 vs. −1.7 to 6.5 pmol L^−1^) and Δ sCr (GT38 vs. LT38: 10 vs. 2 μmol L^−1^, 95% CI of difference 6 to 15 vs. −3 to 6 μmol L^−1^). This represented effect sizes of 1.9 and 1.6 for higher *T*_c_ group (GT38 vs. LT38) on copeptin and sCr, respectively. In contrast, normetanephrine increased in both groups (GT38 vs. LT38: 275 vs. 169 pmol L^−1^, 95% CI of difference 167–383 vs. 54–285 pmol L^−1^, effect size 0.9), cortisol decreased in both groups (GT38 vs. LT38: −247 vs. −354 nmol L^−1^, 95% CI of difference −357 to −136 vs. −472 to −236 nmol L^−1^, effect size 0.9) and osmolality did not change in either group (GT38 vs. LT38: 2 vs. −1 mosm kg^−1^, 95% CI of difference −2 to 6 vs. −5 to 3 mosm kg^−1^, effect size 0.7). In those in whom sweat samples were available, GT38 (*n* = 6) vs. LT38 (*n* = 4) tended to have a higher sweat sodium but this did not reach statistical significance (42.5 vs. 28.8 mmol L^−1^, 95% CI of difference 28.1 to −0.6 mmol L^−1^, effect size 1.5).

## Discussion

This is the first study to investigate relationships between copeptin and *T*_c_ responses. The principal finding of the study was that both copeptin and sCr discriminated groups by *T*_cMax_, showing significant changes for GT38 but not LT38. Despite a rise in normetanephrine across the assault exposure and evidence of a strong relationship between copeptin and osmolality, changes in cortisol, normetanephrine and osmolality did not reflect *T*_cMax_ as well as copeptin and sCr. As described in the introduction, copeptin has been found to be of prognostic value in various conditions such as sepsis (Seligman et al. 2008), septic and haemorrhagic shock (Morgenthaler et al. 2007), in lower respiratory tract infections (Müller et al. 2007), heart failure (Stoiser et al. 2006) and myocardial infarction (Marston et al. 2016). We report for the first time an initial association between increased copeptin and higher physiological strain under thermal stress.

During submaximal exercise in the heat, osmolality is known to increase in proportion to the reduction in total body water from evaporative sweating. This has been shown to impair mechanisms involved in the dissipation of body heat, even in the presence of high aerobic fitness or advanced acclimatisation status (Cadarette et al. 1984; Fortney et al. 1984; Sawka et al. 1983). Osmolality and AVP couple closely following submaximal exercise in a hot laboratory environment—despite varying hydration, exercise intensity and *T*_c_ response (Montain et al. 1997)—and before and after prolonged submaximal running in a hot field environment (Mudambo et al. 1997). Significant relationships have also been reported between copeptin and osmolality, both at rest (Szinnai et al. 2007) and after prolonged endurance running (Bracher et al. 2012). In the present study, the strong association between copeptin and osmolality POST (*r* = 0.70, *P* = 0.004) indicated a persistent relationship between AVP and osmolality in the warm field environment.

Although threshold osmolality for AVP release shows marked inter-individual variation and may rarely exceed 290 mosm kg^−1^ H_2_O, it is expected that osmotic stimulus to copeptin release existed in the majority of volunteers during the assault. Comparison of the regression lines for copeptin and osmolality (PRE vs. POST) however suggested a change in the nature of the relationship between copeptin and osmolality over the course of the exposure. The increase in copeptin was 0.83 pmol L^−1^ per unit increase in osmolality PRE, but almost doubled to 1.64 pmol L^−1^ per mosm kg^−1^ H_2_O POST. The PRE relationship was not significant but did represent an important tendency (*r* = 0.46, *P* = 0.08, *n* = 15), where it is speculated that rise in *T*_c_ increased the sensitivity for osmotic release of AVP/copeptin from PRE to POST. This would be consistent with laboratory observations of the effect of passive heating upon the relationship between AVP and osmolality (Takamata et al. 1995), whereby *T*_c_ rise of up to 1.0 °C progressively augmented AVP secretion above a threshold osmolality of 290 mosm kg^−1^. This mechanism could also account for increases in copeptin without significant elevation in osmolality observed for the GT38 group. The relationship of copeptin response to *T*_c_ excursion is further supported by the strong correlation shown between Δ copeptin and AUC-Tc37 (*r* = 0.78, *P* = 0.0007).

The finding that sweat sodium tended to be higher in GT38 argues against osmotic stimulation being the only significant driver of copeptin response in this study, as relatively higher sweat tonicity would diminish the rise in serum osmolality from hypotonic sweat losses. Rather, our results confirm the advancing acclimatisation status of the volunteers—with group sweat sodium measures being in the lower part of the expected range—and raise the possibility that copeptin responses may have reflected differences in the rate of heat adaptation between GT38 and LT38, with LT38 having lesser copeptin response while appearing better adapted both by *T*_c_ and sweat sodium concentration.

An alternate or complementary explanation for the reported findings is that true ‘non-osmotic’ secretion of AVP and copeptin occurred. When Maresh et al. (2004) assessed the effect of drinking ad libitum upon osmolality and AVP responses during low-intensity exercise in a warm laboratory environment, significant AVP release was evident despite unchanged osmolality (~ 287 mosm kg^−1^ H_2_O) and only 1% loss of body mass across a 90 min exposure. It was suggested that non-osmotic peripheral secretion of AVP occurred secondary to activation of the sympathetic nervous system. Mellor et al. (2015) showed a rise in copeptin with exercise under progressively more stressful conditions during a high altitude trek. As for AVP this occurred independently of osmolality, but whereas increasing altitude (and physiological stress) was shown to have an effect on copeptin response, the corollary was not significant for AVP. This disparity may have reflected known difficulties in the assay of AVP; it is also possible that copeptin better reflected non-osmotic stimuli, including headache, nausea, anxiety and stress from exertion in conditions of hypobaric hypoxia.

In the present study, assay of plasma free normetanephrine was undertaken to provide information about changes in sympathetic outflow and norepinephrine metabolism (Deutschbein et al. 2010; Woods et al. 2017). As a means of assessing sympathoadrenal activation during exercise in field conditions, this approach may have avoided certain pitfalls associated with norepinephrine measurement, relating to changes in posture or stress stimuli proximal to collection. Whereas an interaction between time and *T*_c_ group existed for copeptin responses, no such relationship was demonstrated for the normetanephrine or cortisol during the exposure. This finding would favour a genuine enhanced secretion of AVP/copeptin in consequence of greater relative thermal strain, rather than from more general stress associated with sympathoadrenal stimulation. Indeed, while copeptin has been found to rise after prolonged endurance exercise (over 160 km endurance races) (Hew-Butler et al. 2011) the GPS and cortisol data show that the exercise undertaken in our study was far from intense, and unlikely to explain the changes seen. And despite various other physical discomforts and psychological stressors that might be expected to serve as non-osmotic stimuli during simulated field combat, a clear interaction between training and *T*_c_ group was evident. These observations strengthen the suggestion that copeptin may be of use in characterising physiological strain under heat stress in particular and that the relationship warrants further investigation.

In addition to *T*_c_ rise, thermal strain encompasses the cardiovascular thermo-effector responses to exertion in a warm environment, which must meet the competing needs of increased blood supply to exercising muscle (metabolic demand) and skin (thermoregulation). These changes occur at the cost of blood flow to the kidneys and acute kidney injury is a well-recognised complication of severe heat illness (Leon and Bouchama 2015). The rise in sCr demonstrated by GT38 may reflect greater thermoregulatory strain relative to LT38, and it is possible that reduced renal blood flow and glomerular filtration rate (GFR) contributed to the differential copeptin response between the two groups. The robust correlation between copeptin and sCr supports this alternate hypothesis. Interestingly, recent work using the synthetic analogue of AVP, desmopressin, has also suggested a potential role for AVP in inducing chronic kidney disease following recurrent heat stress in mice (Roncal-Jimenez et al. 2016). It is also therefore possible that what we have seen in association with GT38 could reflect a pathophysiological mechanism to cause impaired kidney function in humans with thermal stress. Chronic kidney disease is epidemic among workers subject to dehydration in hot agricultural environments (Bodin et al. 2016), with one recent investigation showing an increased risk of acute kidney injury from higher thermal strain during shifts in the field (Moyce et al. 2017). The mean maximum *T*_c_ in that larger sample was 38 °C.

While copeptin measurement presently requires the use of laboratory-based assay platforms, no more than 50 μL of sample is required, no extraction step is needed and results can be ready in as little as 30 min (Morgenthaler 2012). This suggests that point-of-care assay will be feasible in the near future. In the meantime, the stability of the molecule (stable for ≥ 7 days at room temperature) reduces the logistics required for sample transfer from field/working environments to the laboratory, such that copeptin assay could be used to further investigate cumulative physiological strain in relation to next-day working practices and prevention of heat-related illnesses.

We acknowledge several limitations to our study. In the austere environment in which it was conducted we were unable to accurately measure changes in plasma volume. In addition to increased production and impaired clearance, a reduction in plasma volume from pre to post could further account for elevated plasma copeptin and sCr. However, it would seem unlikely to explain the 400–500% differences in copeptin and sCr responses between GT38 and LT38. In addition, the temporal relationships of the reported biochemical parameters to other aspects of the field assault should be addressed. In order to undertake study measures in a field setting with the necessary standardisation and control, the investigators co-located with volunteers during the ‘live-firing’ phase of a busy infantry training exercise. Geographical and time constraints necessitated pre sampling being completed 5 h before the start of the field assault, though volunteers did not engage in strenuous physical exertion and were exposed to much cooler environmental temperatures in the intervening period (3.00 a.m.–8.00 a.m.), such that peak *T*_c_ remained below the level observed during the field assault. The advantage of sampling volunteers shortly after rising was that pre results could be regarded as true ‘steady state’ values, which would otherwise be impossible to obtain during a military deployment of this kind. The subsequent change in copeptin reflected the stressors of the intervening period rather than circadian rhythmicity, as copeptin does not synchronise with the light–dark cycle in healthy humans (Darzy et al. 2010). While we report greater rises in copeptin in GT38 vs. LT38, suggesting that copeptin reflects thermal strain, there were no cases of heat illness in our cohort. As the participants followed an appropriate period of acclimatisation and exertion levels were modest this is as expected. Further, it would have been unethical to specifically aim to induce heat casualties. Cardiovascular strain—which may also contribute to collapse during thermal stress—was not measured directly and further work is required to determine how this and other relevant factors, such as inflammatory responses, relate to copeptin and whether a single measurement of copeptin might have diagnostic/prognostic value at higher levels of heat strain and in heat illness cases.

In conclusion, the change in copeptin reflected *T*_c_ response according to the occupational and physiological threshold of *T*_c_ = 38 °C, with over a fourfold greater rise in GT38 vs. LT38. The post-exercise coupling between copeptin and osmolality appeared to persist in the warm training environment, but may have been modified by changes in the osmotic sensitivity of AVP/copeptin release due to heating, as well as by changes in GFR. It is suggested that copeptin may be of future utility in assessing health risks associated with working under thermal stress, with potential to reflect global physiological strain and possibly also pathophysiological mechanisms contributing to heat-related morbidity. A point-of-care assay for copeptin would afford opportunity for in-field evaluation of a potential marker of integrated physiological strain alongside more conventional thermal stress monitoring. Further work is required to delineate the copeptin response to greater thermal stress both in health and ultimately in heat-related illnesses, as well as the effects of hydration and acclimatisation status.

## Abbreviations

ANOVA: Analysis of variance
AUC-Tc37: Area under the core body temperature–time curve (baseline *T*_c_ 37 °C)
AVP: Arginine vasopressin
LT38: Volunteers with maximum *T*_c_ observed ≤ 38.0 °C
GFR: Glomerular filtration rate
GPS: Global positioning system
GT38: Volunteers with maximum observed *T*_c_ > 38.0 °C
PRE: Before assault exposure
POST: After assault exposure
sCr: Serum creatinine
SD: Standard deviation
*T*_c_: Core body temperature
*T*_cMax_: Maximum observed *T*_c_
WBGT: Wet bulb globe temperature

## References

[CR1] Armstrong LE, Casa DJ, Millard-Stafford M, Moran DS, Pyne SW, Roberts WO (2007). American College of Sports Medicine position stand. Exertional heat illness during training and competition. Med Sci Sports Exerc.

[CR2] Bodin T, Garcia-Trabanino R, Weiss I (2016). Intervention to reduce heat stress and improve efficiency among sugarcane workers in El Salvador: phase 1. Occup Environ Med.

[CR3] Bracher A, Knechtle B, Gnadinger M (2012). Fluid intake and changes in limb volumes in male ultra-marathoners: does fluid overload lead to peripheral oedema?. Eur J Appl Physiol.

[CR4] Cadarette BS, Sawka MN, Toner MM (1984). Aerobic fitness and the hypohydration response to exercise-heat stress. Aviat Space Environ Med.

[CR5] Cheuvront SN, Kenefick RW (2014). Dehydration: physiology, assessment, and performance effects. Compr Physiol.

[CR6] Christ-Crain M, Fenske W (2016). Copeptin in the diagnosis of vasopressin-dependent disorders of fluid homeostasis. Nat Rev Endocrinol.

[CR7] Commission for Thermal Physiology of the International Union of Physiological Sciences (1987). Glossary of thermal physiology Pflügers. Arch.

[CR8] Darzy KH, Dixit KC, Shalet SM (2010). Circadian secretion pattern of copeptin, the C-terminal vasopressin precursor fragment. Clin Chem.

[CR9] Deutschbein T, Unger N, Jaeger A (2010). Influence of various confounding variables and storage conditions on metanephrine and normetanephrine levels in plasma. Clin Endocrinol.

[CR10] Fortney SM, Wenger CB, Bove JR (1984). Effect of hyperosmolality on control of blood flow and sweating. J Appl Physiol.

[CR11] Garzon-Villalba XP, Mbah A, Wu Y, Hiles M, Moore H, Schwartz SW, Bernard TE (2016). Exertional heat illness and acute injury related to ambient wet bulb globe temperature. Am J Ind Med.

[CR12] Gonzalez-Alonso J, Teller C, Andersen SL (1999). Influence of body temperature on the development of fatigue during prolonged exercise in the heat. J Appl Physiol.

[CR13] Greisenegger S, Segal HC, Burgess AI (2015). Copeptin and long-term risk of recurrent vascular events after transient ischemic attack and ischemic stroke: population-based study. Stroke.

[CR14] Hew-Butler T, Hoffman MD, Stuemple KJ (2011). Changes in copeptin and bioactive vasopressin in runners with and without hyponatraemia. Clin J Sport Med.

[CR15] Katan M, Morgenthaler N, Widmer I (2008). Copeptin, a stable peptide derived from the vasopressin precursor, correlates with the individual stress level. Neuroendocrino Lett.

[CR16] Katan M, Müller B, Christ-Crain M (2008). Copeptin: a new and promising diagnostic and prognostic marker. Crit Care.

[CR17] Leon LR, Bouchama A (2015). Heat stroke. Compr Physiol.

[CR18] Maresh CM, Gabaree-Boulant CL, Armstrong LE (2004). Effect of hydration status on thirst, drinking and related hormonal responses during low-intensity exercise in the heat. J Appl Physiol.

[CR19] Marston NA, Shah KS, Mueller C (2016). Serial sampling of copeptin levels improves diagnosis and risk stratification in patients presenting with chest pain: results from the CHOPIN trial. Emerg Med J.

[CR20] Meade RD, Poirier MP, Flouris AD, Hardcastle SG, Kenny GP (2016). Do the threshold limit values for work in hot conditions adequately protect workers?. Med Sci Sports Exerc.

[CR21] Mellor AJ, Boos CJ, Ball S, Burnett A, Pattman S, Redpath M, Woods DR (2015). Copeptin and arginine vasopressin at high altitude: relationship to plasma osmolality and perceived exertion. Eur J Appl Physiol.

[CR22] Montain SJ, Laird JE, Latzka WA (1997). Aldosterone and vasopressin responses in the heat: hydration level and exercise intensity. Med Sci Sports Exerc.

[CR23] Morgenthaler N (2012). Copeptin—the steady and reliable copilot of vasopressin. Regul Pept.

[CR24] Morgenthaler NG, Struck J, Alonso C, Bergmann A (2006). Assay for the measurement of copeptin, a stable peptide derived from the precursor of vasopressin. Clin Chem.

[CR25] Morgenthaler NG (2007). Copeptin, a stable peptide of the arginine vasopressin precursor, is elevated in hemorrhagic and septic shock. Shock.

[CR26] Moyce S, Mitchell D, Armitage T (2017). Heat strain, volume depletion and kidney function in California agricultural workers. Occup Environ Med.

[CR27] Mudambo KSMT, Coutie W, Rennie MJ (1997). Plasma arginine vasopressin, atrial natriuretic peptide and brain natriuretic peptide responses to long-term field training in the heat: effects of fluid ingestion. Eur J Appl Physiol.

[CR28] Müller B, Morgenthaler N, Stolz D (2007). Circulating levels of copeptin, a novel biomarker, in lower respiratory tract infections. Eur J Clin Investig.

[CR29] Noakes TD (2008). A modern classification of the exercise-related heat illnesses. J Sci Med Sport.

[CR30] Rhind SG, Gannon GA, Shek PN (1999). Contribution of exertional hyperthermia to sympathoadrenal-mediated lymphocyte subset redistribution. J Appl Physiol.

[CR31] Roncal-Jimenez CA, Milagres T, Andres-Hernando A (2016). Effects of exogenous desmopressin on a model of heat stress nephropathy in mice. Am J Physiol Renal Physiol.

[CR32] Sawka MN, Toner MM, Francesconi RP (1983). Hypohydration and exercise: effects of heat acclimation, gender, and environment. J Appl Physiol.

[CR33] Seligman R, Papassotiriou J, Morgenthaler NG (2008). Copeptin, a novel prognostic biomarker in ventilator-associated pneumonia. Crit Care.

[CR34] Sladek CD, Johnson AK (2013). Integration of thermal and osmotic regulation of water homeostasis: the role of TRPV channels. Am J Physiol Regul Integr Comp Physiol.

[CR35] Stoiser B, Mortl D, Hulsmann M (2006). Copeptin, a fragment of the vasopressin precursor, as a novel predictor of outcome in heart failure. Eur J Clin Investig.

[CR36] Sun H, Sun T, Ma B (2015). Prediction of all-cause mortality with copeptin in cardio-cerebrovascular patients: a meta-analysis of prospective studies. Peptides.

[CR37] Szinnai G, Morgenthaler NG, Berneis K (2007). Changes in plasma copeptin, the C-terminal portion of arginine vasopressin during water deprivation and excess in healthy subjects. J Clin Endocrinol Metab.

[CR38] Takamata A, Mack GW, Stachenfeld NS (1995). Body temperature modification of osmotically induced vasopressin secretion and thirst in humans. Am J Physiol.

[CR39] Velho G, El Boustany R, Lefèvre G (2016). Plasma copeptin, kidney outcomes, ischemic heart disease, and all-cause mortality in people with long-standing type 1 diabetes. Diabetes Care.

[CR40] Wallace RF, Kriebel D, Punnett L, Wegman DH, Wenger CB, Gardner JW, Gonzalez RR (2005). The effects of continuous hot weather training on risk of exertional heat illness. Med Sci Sports Exerc.

[CR41] Woods DR, O’Hara JP, Boos CJ, Hodkinson PD, Tsakirides C, Hill NE, Jose D, Hawkins A, Phillipson K, Hazlerigg A, Arjomandkhah N, Gallagher L, Holdsworth D, Cooke M, Green ND, Mellor A (2017). Markers of physiological stress during exercise under conditions of normoxia, normobaric hypoxia, hypobaric hypoxia, and genuine high altitude. Eur J Appl Physiol Mar.

[CR42] Wyndham CH, Strydom NB, Morrison (1965). Criteria for physiological limits for work in heat. J Appl Physiol.

[CR43] Yeoh WK, Lee JKW, Lim HY, Gan CW, Liang W, Tan KK (2017). Re-visiting the tympanic membrane vicinity as core body temperature measurement site. PLoS One.

